# The Primo Vascular System as a Biophysical Network: A Theoretical Framework for Meridian Function and Autonomic Regulation

**DOI:** 10.7759/cureus.115471

**Published:** 2026-08-30

**Authors:** Daniele Gramolelli

**Affiliations:** 1 Osteopathic Medicine, Istituto Europeo per la Medicina Osteopatica, Genova, ITA

**Keywords:** acupuncture meridians, autonomic regulation, bioelectric signaling, biophysical communication, connective tissue, extracellular matrix, interstitial fluid, mechanotransduction, primo vascular system, tissue impedance

## Abstract

The primo vascular system (PVS) has been proposed as a distinct anatomical network composed of primo vessels and primo nodes distributed within and around blood vessels, lymphatic structures, organs, and connective tissues. Although its biological significance remains incompletely established, experimental observations have raised the possibility that the PVS may participate in forms of interstitial, cellular, or biophysical communication. This technical report presents a conceptual framework integrating the PVS with connective tissue and extracellular matrix organization, interstitial fluid dynamics, and bioelectrical phenomena. Within the proposed model, mechanical or acupuncture-related stimulation may induce local tissue deformation, ionic redistribution, fluid shifts, and changes in electrical properties. These effects could propagate preferentially through anatomically and biophysically heterogeneous tissues, while the PVS is considered as a possible, but currently unconfirmed, coupling substrate. The framework further explores the hypothesis that such preferential pathways could support oscillatory or standing-wave-like behavior and examines whether this concept may contribute to future mechanistic interpretations of meridian organization and acupuncture-point phenomena. Importantly, the model does not establish an anatomical equivalence between the PVS and traditional meridians, nor does it demonstrate that standing-wave phenomena occur within the PVS. Rather, these relationships are presented as testable hypotheses. The framework generates experimentally accessible predictions involving tissue electrical properties, impedance, interstitial dynamics, spatial signal propagation, and autonomic or neurovascular responses. This model is intended to provide a structured basis for experimental investigation of the PVS and its potential role in integrative biophysical communication.

## Introduction

The primo vascular system (PVS) has been described as a putative anatomical network composed of primo vessels and primo nodes distributed within and around blood vessels, lymphatic structures, internal organs, connective tissues, and body cavities. Since its original description, several experimental studies have reported structures compatible with the PVS in different animal models and anatomical regions. However, its morphology, physiological significance, functional continuity, and relevance to human biology remain incompletely established.

Interest in the PVS has been partly driven by the possibility that it may represent a previously underrecognized biological interface involved in interstitial transport, cellular communication, tissue signaling, or other forms of long-range physiological integration. Some authors have also proposed a possible relationship between the PVS and the traditional meridian system described in East Asian medicine. This association remains hypothetical and should not be interpreted as evidence that acupuncture meridians correspond anatomically to the PVS.

At the same time, contemporary research on connective tissue, extracellular matrix organization, mechanotransduction, interstitial fluid dynamics, and endogenous bioelectrical signaling has expanded the understanding of how mechanical and electrical information may propagate across tissues. Fascia and extracellular matrix structures are increasingly recognized as dynamic biological environments capable of transmitting mechanical forces, modifying local ionic conditions, influencing cellular behavior, and interacting with vascular and neural systems.

These observations raise the possibility that the PVS, if confirmed as a functionally relevant network, may interact with broader connective-tissue and interstitial systems rather than operate as an isolated anatomical structure. Such an interaction could provide a framework for investigating whether local mechanical or acupuncture-related stimulation produces spatially organized changes in tissue impedance, fluid distribution, bioelectrical activity, autonomic regulation, or neurovascular responses.

The purpose of this technical report is to present a hypothesis-generating biophysical framework integrating the PVS with fascia, extracellular matrix, interstitial fluids, bioelectrical signaling, and preferential tissue propagation. The framework explicitly distinguishes biological processes that are established or experimentally supported from components that remain hypothetical. In particular, functional coupling through the PVS, correspondence with traditional meridian organization, and standing-wave-like spatial behavior are not presented as demonstrated mechanisms. Rather, these elements are formulated as experimentally testable hypotheses intended to guide future anatomical, biophysical, and physiological investigation.

## Technical report

Anatomical and biophysical basis of the proposed framework

The proposed model is based on the convergence of three distinct biological domains: the reported anatomy of the primo vascular system (PVS), the structural and mechanical properties of connective tissue and extracellular matrix (ECM), and the capacity of biological tissues to exhibit electrochemical and bioelectrical phenomena. These domains should be considered separately before their potential interaction is discussed. Primo vessels and primo nodes have been reported in several anatomical compartments, including organ surfaces, lymphatic vessels, structures associated with the nervous system, blood vessels, and fascia-like tissues [1-5]. Structures described as PVS have also been reported in the human umbilical cord and placenta [6]. Nevertheless, the distribution, continuity, physiological function, and broader relevance of the PVS in humans remain incompletely characterized, and methodological difficulties in identifying and distinguishing these structures have repeatedly been recognized in the literature [2,5,7]. Accordingly, anatomical observations of the PVS provide a potential structural basis for investigation but do not, by themselves, establish a role in long-range biological communication or correspondence with traditional meridian pathways. A further methodological limitation concerns the identification of PVS structures themselves. No single currently established histological or immunohistochemical marker can independently be regarded as diagnostic of a primo vessel. Reliable identification therefore requires converging morphological, histological, ultrastructural, and anatomical criteria together with careful exclusion of conventional lymphatic vessels, small blood vessels, nerves, connective-tissue strands, and fibrinous material. Consequently, attribution of physiological properties or signal-transmission phenomena to the PVS should require prior independent morphological confirmation of the structures being investigated.

Connective tissue and the ECM form mechanically responsive environments capable of transmitting forces and mediating cellular mechanotransduction [8,9]. Interstitial fluid movement contributes to transport within soft tissues and interacts dynamically with the extracellular matrix [10,11]. Mechanical loading can also interact with ionic and electrochemical processes within extracellular matrices [9-12], although the magnitude and biological relevance of such effects depend strongly on tissue composition and experimental conditions. In parallel, endogenous bioelectrical signaling generated through ion channels, pumps, and transmembrane voltage gradients is an established component of cellular and tissue regulation [13]. These mechanisms should not be interpreted as evidence of electrical conduction through the PVS; rather, they define a broader biophysical environment within which a potential contribution of PVS structures may be investigated.

Acupuncture-related mechanical stimulation also provides an experimental example of interaction between an externally applied stimulus and connective tissue. Needle manipulation has been shown to induce connective-tissue deformation and fibroblast cytoskeletal responses consistent with mechanotransduction [14]. Anatomical studies have additionally reported associations between some acupuncture locations and connective-tissue planes, although these observations do not establish that connective tissue represents a unique anatomical substrate of the traditional meridian system [15].

The framework proposed below therefore does not require the PVS to function as an isolated conducting network. Instead, it asks whether PVS structures could participate in, modulate, or spatially organize processes occurring within a larger fascia-ECM-interstitial environment. This distinction is central to the model because it separates established tissue biology from the additional and currently hypothetical functional contribution attributed to the PVS [16].

Proposed integrated fascia-PVS framework

The proposed framework considers the PVS not as an isolated anatomical system but as a possible component of a broader connective-tissue and interstitial environment. Local mechanical or acupuncture-related stimulation can deform connective tissue and extracellular matrix and initiate mechanotransductive responses [9,14]. Such stimulation may also influence local fluid distribution and physicochemical conditions within the interstitial environment [10,11]. These phenomena constitute the established or biologically plausible first level of the proposed model.

Mechanical deformation, fluid movement, ionic conditions, cellular mechanotransduction, and endogenous bioelectrical processes may interact within living tissues [9-13]. Their spatial distribution would not necessarily be uniform because biological tissues differ in geometry, extracellular-matrix organization, fluid content, vascularization, innervation, and electrical properties. Experimental work on acupuncture needling has, for example, demonstrated tissue responses dependent on connective-tissue architecture [14,15]. This observation does not demonstrate the existence of meridian-specific propagation but supports investigating whether structurally heterogeneous tissues generate preferential patterns of response.

Within this broader environment, the PVS is proposed as a potential additional coupling substrate. If primo vessels and primo nodes form functionally interconnected pathways, they could theoretically interact with the surrounding extracellular matrix and interstitial fluids and contribute to the spatial organization of biophysical signals. Previous literature has explicitly discussed possible relationships among fascia, the PVS, and meridian concepts, but these relationships remain theoretical [16]. Direct evidence demonstrating long-range mechanical, electrical, electrochemical, or fluid-mediated signal propagation specifically through the PVS remains insufficient; consequently, this component of the model should be regarded as a hypothesis rather than an established mechanism [5,7].

The conceptual sequence proposed in this report is illustrated in Figure 1. Local stimulation is followed by mechanical and physicochemical changes within fascia and extracellular matrix, generation or modification of biophysical signals, and potentially nonuniform propagation through heterogeneous tissue environments. Fascial continuity and interstitial fluid dynamics represent established biological substrates [8-12], whereas participation of the PVS in spatial signal organization represents the principal additional hypothesis requiring direct experimental validation.

**Figure 1:**
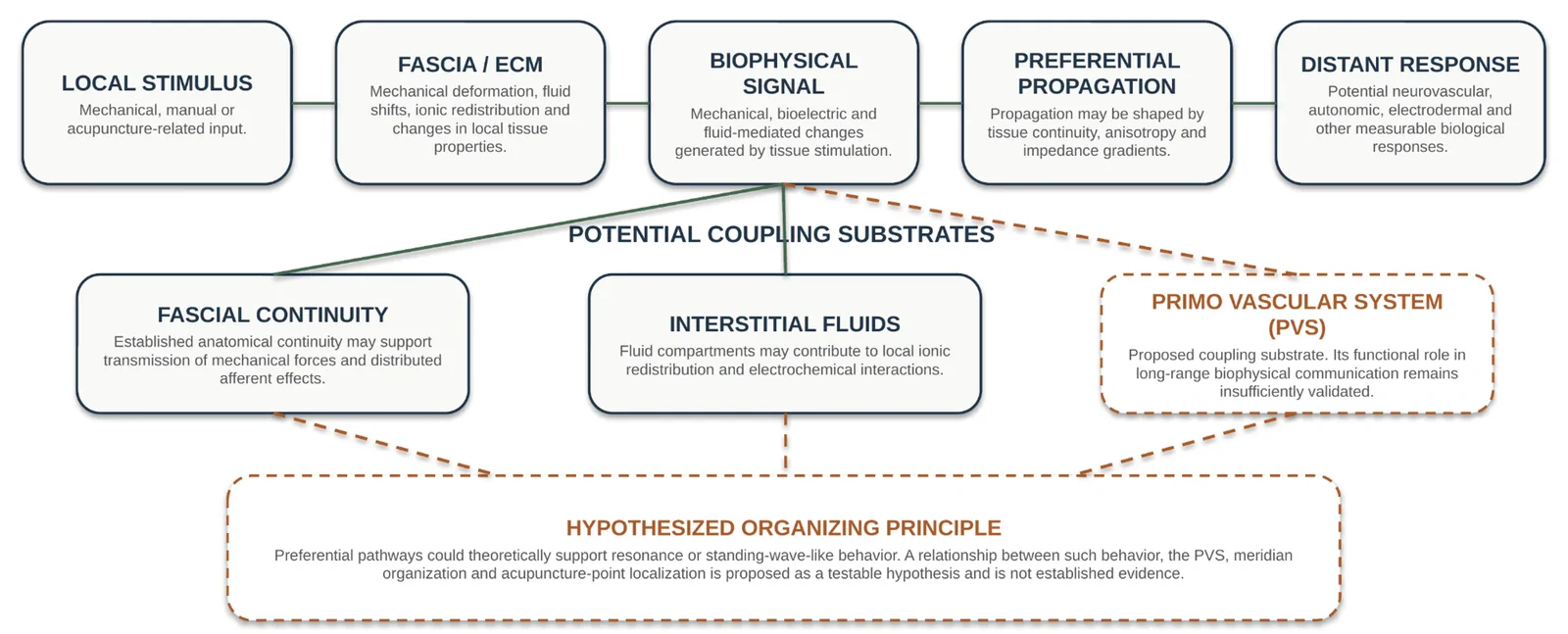
Hypothetical Integrated Fascia-PVS Framework for Biophysical Communication Solid green arrows indicate relationships grounded in established anatomical or biophysical principles, whereas dashed orange arrows and borders indicate proposed or insufficiently validated relationships. The framework integrates local stimulation, fascia and extracellular matrix (ECM), biophysical signaling, preferential tissue propagation, and potential distant biological responses. Fascial continuity and interstitial fluids are considered plausible coupling substrates, while involvement of the primo vascular system (PVS) is proposed as a hypothesis requiring direct experimental validation. The framework does not establish that the PVS corresponds to traditional meridians.

Hypothesized resonance and standing-wave behavior

Biological tissues constitute heterogeneous mechanical, fluid-dynamic, electrochemical, and bioelectrical environments [8-13]. They are also continuously exposed to periodic physiological inputs arising from cardiac, respiratory, vascular, neural, and cellular activity. Within the present framework, differences in tissue geometry, mechanical properties, fluid distribution, electrical characteristics, and structural coupling are proposed as variables that could influence the spatial behavior of oscillatory biological signals.

A further and explicitly speculative extension of the framework is that, under appropriate boundary and coupling conditions, interactions between periodic inputs and preferential propagation pathways could theoretically produce standing-wave-like spatial patterns characterized by relatively stable regions of higher and lower signal amplitude. This report does not assume that such patterns have been demonstrated in the PVS. No specific biological propagation medium, boundary condition, wavelength, frequency range, propagation velocity, or energy source can presently be assigned to this hypothesis, because these physical parameters have not been experimentally established for the PVS or for a putative fascia-interstitial-PVS oscillatory system. Accordingly, the standing-wave-like concept should not be interpreted as a quantitatively specified physical model at the present stage. Experimental support for this component would require demonstration of reproducible frequency-dependent spatial patterns together with measurable phase relationships, propagation characteristics, attenuation, and sensitivity to defined boundary conditions. Failure to demonstrate such properties would argue against the standing-wave component of the framework. Likewise, the literature cited here does not establish that acupuncture points represent nodes or antinodes of biological standing waves.

The value of this component of the framework therefore lies in its experimental testability rather than in existing proof. If reproducible oscillatory spatial organization exists, standardized stimulation should produce stable and measurable differences in parameters such as tissue electrical impedance, surface electrical potential, mechanical displacement, microvascular responses, interstitial dynamics, or autonomic variables. Existing investigations of electrical characteristics at acupuncture points and meridians have produced suggestive but methodologically heterogeneous results and have not conclusively demonstrated that these locations possess unique electrical properties [17]. Consequently, electrical impedance should be treated as one candidate measurement among several rather than as evidence for the model itself.

An oscillatory model would further predict that changing relevant experimental variables, such as stimulation frequency, intensity, tissue mechanical tension, or boundary conditions, should modify the location or magnitude of any reproducible spatial pattern. Conversely, failure to demonstrate stable spatial organization under controlled conditions would weaken this component of the framework. Accordingly, standing-wave behavior is not proposed here as an established mechanism of meridian function. It is presented as a falsifiable biophysical hypothesis that may guide experimental investigation of spatial organization within a fascia-interstitial fluid-PVS framework.

Relationship with meridian and acupuncture-point organization

A possible relationship between the PVS and the meridian system described in traditional East Asian medicine has been discussed since the original Bong-Han hypothesis and its subsequent re-examination in modern PVS research [1,2]. Several authors have proposed that the PVS could provide an anatomical substrate related to acupuncture meridians [1,16]. However, critical reviews have concluded that available evidence remains insufficient to establish a direct correspondence between documented PVS structures and traditional meridian trajectories [18].

The present framework therefore does not interpret meridians as discrete anatomical conduits represented exclusively by the PVS. Instead, it explores whether phenomena historically organized within the concept of meridians could, in part, reflect an emergent spatial organization involving multiple biological substrates, including connective-tissue architecture, interstitial fluids, vascular and neural structures, local electrical properties, and, hypothetically, the PVS.

This interpretation is consistent with previous investigations of connective tissue and acupuncture-point organization. Langevin and Yandow reported a substantial anatomical association between acupuncture-point locations and intermuscular or intramuscular connective-tissue planes in the human arm [15]. Other authors have subsequently explored fascia-based models of acupuncture organization [16]. These observations provide a rationale for examining spatial tissue organization but do not demonstrate that classical meridians correspond anatomically to fascia, the PVS, or any single biological structure.

Acupuncture points may similarly be investigated as candidate regions in which several tissue characteristics converge. Potential variables include connective-tissue architecture, innervation, vascularization, interstitial composition, electrical impedance, and mechanical responsiveness. Evidence concerning electrical characteristics is particularly important to interpret cautiously: a systematic review found some studies reporting lower electrical resistance or impedance at acupuncture points or along meridian-related regions, but methodological limitations prevented a definitive conclusion that these locations are electrically distinct [17].

Within the standing-wave component of the present model, acupuncture locations could theoretically coincide with regions displaying relatively stable differences in oscillatory amplitude or coupling. This possibility is deliberately presented as speculative. No equivalence between acupuncture points and biological nodes or antinodes is assumed.

The model consequently shifts the principal experimental question from whether the PVS is the meridian system to whether PVS structures participate in a multimodal biological network exhibiting reproducible spatial organization. This distinction permits anatomical, mechanical, electrical, vascular, neural, and interstitial variables to be investigated independently rather than requiring a priori acceptance of a one-to-one PVS-meridian correspondence. Demonstrating reproducible spatial relationships among these variables would support specific components of the framework; failure to identify such relationships would progressively constrain or refute them.

Testable predictions and experimental validation

The proposed framework generates a series of experimentally testable predictions. Rather than attempting to validate the model as a whole, future investigations should examine individual components independently and determine whether predicted anatomical, spatial, and physiological relationships can be reproduced under controlled conditions. First, if preferential biophysical pathways exist within connective and interstitial tissues, standardized local stimulation should generate reproducible spatial organization rather than completely uniform responses. Candidate measurements could include tissue electrical impedance, surface electrical potentials, mechanical displacement or strain, interstitial fluid dynamics, microvascular perfusion, and autonomic variables. Because each of these measures can be influenced by numerous physiological and technical factors, repeated measurements, standardized environmental conditions, and appropriate control locations would be required. Existing limitations in electrical studies of acupuncture locations illustrate the importance of controlling skin hydration, electrode pressure, measurement geometry, and other potential confounders [17].

Second, the proposed contribution of the PVS requires direct anatomical and functional investigation. Studies should determine whether independently identified primo vessels and primo nodes exhibit reproducible spatial relationships with connective-tissue planes, vascular or neural structures, interstitial compartments, or regions displaying distinctive biophysical properties. Histological and immunohistochemical identification should be combined, where technically possible, with imaging and physiological measurement. Existing PVS research demonstrates that identification itself is technically challenging, and therefore morphological confirmation should precede attribution of any measured physiological phenomenon to the PVS [2,5,7].

Third, functional participation of the PVS cannot be established through anatomical colocalization alone. If the PVS contributes to signal transmission or spatial organization, engagement of PVS-associated structures should be associated with reproducible changes in measurable physiological variables. Stronger evidence would require demonstrating that selective modification, interruption, or differential engagement of those structures systematically alters the observed response.

Fourth, the standing-wave component generates a distinct prediction. If oscillatory interactions contribute to spatial organization, reproducible regions of relatively higher and lower signal amplitude should emerge under defined stimulation conditions. Their positions should demonstrate greater stability than expected from measurement variability or chance and should change predictably when stimulation frequency, intensity, mechanical conditions, or other relevant boundary parameters are modified. Failure to demonstrate such frequency- or condition-dependent organization would weaken the standing-wave component without necessarily invalidating the remainder of the fascia-PVS framework.

Fifth, any proposed relationship with acupuncture points or meridian trajectories should be tested without assuming correspondence in advance. Electrical, mechanical, vascular, neural, interstitial, and PVS-related variables should first be mapped independently and subsequently compared with predefined acupuncture locations. Blinded assessment, matched non-acupuncture control sites, predefined spatial criteria, and independent replication would be necessary to minimize selection and confirmation bias. This is particularly important because previous research has generated competing anatomical explanations for meridian-related phenomena and has not identified a universally accepted anatomical substrate [15-18].

Finally, the framework predicts that local stimulation could produce measurable distant physiological responses if functionally relevant preferential coupling pathways exist. Such responses could include alterations in autonomic activity, microvascular perfusion, tissue electrical characteristics, or other physiological variables. Observation of a distant response alone, however, would not demonstrate involvement of the PVS because neural, vascular, humoral, connective-tissue, and contextual mechanisms provide alternative explanations. Attribution to the PVS would require evidence that specifically links the response to anatomically verified PVS structures.

Taken together, these experiments would permit progressive evaluation of the framework instead of requiring binary acceptance or rejection of the entire model. Evidence supporting one component would not automatically validate the others. Conversely, failure to confirm specific predictions would permit those components to be modified or rejected. This modular structure preserves the falsifiability of the proposed framework.

## Discussion

The main contribution of the present framework is to reposition the primo vascular system (PVS) within a broader biological context rather than treating it as a self-sufficient anatomical explanation of the traditional meridian system. Previous interpretations have proposed a possible relationship between the PVS and acupuncture meridians, but the available evidence remains insufficient to establish a direct anatomical equivalence [1,2,16,18]. The present model therefore adopts a different approach: the PVS is considered as one possible component of a multimodal tissue environment that also includes connective tissue, extracellular matrix (ECM), interstitial fluids, vascular and neural structures, and endogenous biophysical processes.

This distinction has practical scientific value. If meridian-related phenomena reflect interactions among multiple biological substrates rather than a single dedicated conduit, experimental investigation can move away from the question of whether one anatomical structure “is” a meridian. Instead, measurable variables can be mapped independently and then compared spatially. These may include connective-tissue organization, local mechanical properties, electrical impedance, microvascular behavior, interstitial characteristics, neural distribution, and anatomically verified PVS structures. Such an approach reduces the risk of forcing heterogeneous findings into a predetermined anatomical model.

The framework also separates biological plausibility from mechanistic proof. Mechanotransduction, extracellular-matrix responsiveness, interstitial fluid transport, and endogenous bioelectrical signaling are established biological phenomena [8-14]. Their inclusion in the model therefore provides a plausible biophysical background. By contrast, the functional role attributed to the PVS remains uncertain. Anatomical identification of primo vessels or primo nodes does not demonstrate long-range signaling, preferential transmission, or involvement in distant physiological effects. This separation between established tissue biology and the hypothetical contribution of the PVS is essential to the interpretation of the model.

The same caution applies to acupuncture-point organization. Associations between some acupuncture locations and connective-tissue planes have been reported [15], while investigations of electrical properties at acupuncture points have produced heterogeneous findings [17]. These observations justify further spatial investigation but do not identify a unique anatomical or electrical substrate of acupuncture points. Within the present framework, acupuncture locations are therefore treated as candidate regions where multiple tissue properties might converge rather than as predefined anatomical structures whose biological nature is already known.

The standing-wave hypothesis should be interpreted even more cautiously. It represents the most speculative component of the framework and is included because it generates experimentally falsifiable predictions rather than because standing waves have been demonstrated within the PVS. Biological tissues are heterogeneous and dissipative, and the physical conditions required for stable standing-wave behavior in vivo remain uncertain. The relevance of this hypothesis therefore depends entirely on whether reproducible spatial and frequency-dependent patterns can be demonstrated experimentally.

A potential strength of the model is its modularity. Evidence supporting one component does not validate the entire framework. For example, reproducible differences in electrical impedance or tissue mechanics could exist independently of PVS involvement. Likewise, anatomical confirmation of PVS structures would not establish a role in meridian organization. This modular structure allows individual elements to be supported, modified, or rejected without requiring binary acceptance or rejection of the whole hypothesis.

Limitations of the proposed framework

Several limitations should be acknowledged. First, the anatomical distribution, continuity, physiological function, and relevance of the PVS in humans remain incompletely established [2,5-7]. Although structures described as primo vessels and primo nodes have been reported in several anatomical compartments, their identification remains technically challenging and requires further independent validation. Second, the framework integrates evidence from research domains that differ substantially in methodological quality and level of validation. Findings from connective-tissue biology, extracellular-matrix research, interstitial physiology, bioelectric signaling, acupuncture studies, and PVS research cannot be treated as equivalent evidence. Convergence among these domains supports hypothesis generation but does not demonstrate causal interaction.

Third, the proposed role of the PVS in long-range biophysical communication remains hypothetical. Direct experimental evidence showing that anatomically verified PVS structures transmit mechanical, electrical, electrochemical, or fluid-mediated signals over physiologically meaningful distances is currently insufficient. Fourth, the standing-wave component remains speculative. There is no direct evidence that physiologically relevant standing waves occur within the PVS or that acupuncture points correspond to nodes or antinodes of such patterns. This component should therefore be interpreted strictly as a testable physical hypothesis.

Fifth, a possible relationship between the PVS and traditional meridian organization remains unresolved [16,18]. Spatial correspondence between PVS structures and acupuncture locations, even if demonstrated, would not by itself establish functional or anatomical equivalence with the traditional meridian system. Finally, this technical report presents a conceptual framework rather than an experimentally validated mechanism or clinical protocol. It does not demonstrate treatment efficacy and does not establish causal relationships among fascia, interstitial fluids, the PVS, acupuncture points, and distant physiological responses. Its purpose is to provide a structured and falsifiable basis for future experimental investigation.

## Conclusions

The primo vascular system (PVS) remains an incompletely characterized anatomical and physiological entity, and current evidence does not establish that it represents the anatomical substrate of traditional meridians or that it mediates long-range biophysical communication. The framework proposed in this technical report therefore places the PVS within a broader fascia-extracellular matrix-interstitial environment and treats its possible contribution to spatial organization and signal coupling as a testable hypothesis rather than an established mechanism.

The model further proposes that heterogeneous tissue properties may support preferential propagation of mechanical, electrochemical, or bioelectrical effects and explores standing-wave-like behavior as an explicitly speculative extension of this concept. Its principal value lies in generating falsifiable predictions that can be examined using multimodal anatomical and physiological measurements. Future studies should independently test the contribution of connective tissue, interstitial fluids, verified PVS structures, electrical and mechanical tissue properties, and spatial responses to stimulation. Such an approach may clarify whether the PVS contributes to a broader biophysical network and whether any reproducible relationship exists with acupuncture-point or meridian organization.
